# Physician Well-Being and the Promise of Positive Psychology

**DOI:** 10.31486/toj.22.0109

**Published:** 2023

**Authors:** Mahum Shahid

**Affiliations:** Director of Program Diversity and Early Career Development for Women in Internal Medicine, University of South Dakota Sanford School of Medicine, Sioux Falls, SD

Physician well-being has been gaining more attention across all circuits of physician advocacy given the current state of our health care systems. Physician wellness (well-being), per a systematic review of 78 studies, is defined by quality of life, which includes the absence of ill-being and the presence of positive physical, mental, social, and integrated well-being experienced in connection with activities and environments that allow physicians to develop their full potentials across personal and work-life domains.^1^

Physician burnout has a clear and consistent definition. The World Health Organization *International Classification of Diseases-11* defines burnout as an “occupational syndrome,” cementing the Maslach Burnout Inventory triad of emotional exhaustion, depersonalization, and cynicism or feelings of diminished personal accomplishment as the classic definition of physician burnout.^2^

Mental illness in physicians and burnout, although closely related, are acknowledged as separate entities. Physician suicide rates historically have been higher compared to the general population, with a 2004 meta-analysis reporting rates 1.41 times higher in male physicians and 2.27 times higher in female physicians.^3^ Yet data from the National Violent Death Reporting System showed that physicians who died by suicide were less likely to be under treatment for a mental illness and more likely to have antipsychotics, barbiturates, and benzodiazepines rather than antidepressants in their systems compared to nonphysicians.^4^ Stress, a common occupational hazard in many professions, is a known risk factor of burnout and mental illness. Stress can induce neuroinflammation affecting the brain's hippocampal centers of memory, emotional processing, and cognition.^5^ Resilience**,** the ability to bounce back in the face of adversity, is a protective factor against both burnout and mental illness.^6,7^ Resilience training interventions have been shown to decrease depression, stress, and stress perception in health care workers.^7^ So even if physician burnout is not the result of a resilience deficit but is instead a health system–related issue, resilience training can help reduce some of the impact of chronic workplace stress.

The Dr. Lorna Breen Health Care Provider Protection Act that was signed into law on March 18, 2022, promotes physician wellness and resilience.^8^ This legislation was designed not only to increase awareness but also to establish grants to support mental health and resilience in physicians. As an adjunct to this legislation, physicians and health systems can adopt self-help measures to improve well-being.

## POSITIVE PSYCHOLOGY–BASED INTERVENTIONS

Since 2000, positive psychology-based interventions have emerged as a promising technique to manage stress and to increase psychological resilience and engagement.^9,10^ Positive emotions, purpose, and a sense of achievement can drive the process of flourishing and contribute to individual success.^11^ Most of these interventions are low-cost and time-efficient, with yields comparable to or even better than pharmacologic therapies.^12^ These interventions can be introduced into work routines without the burden of extensive training. Some interventions are targeted toward certain domains of burnout (emotional exhaustion, depersonalization), yet when used at the institutional level, positive psychology-based interventions can support overall physician well-being.

## TIME- AND COST-EFFICIENT EVIDENCE-BASED INTERVENTIONS

Imposter phenomenon—self-doubt and the inability to internalize success—is more prevalent in US physicians than in the US working population according to a 2022 study that compared responses to an item from the Clance Imposter Phenomenon Scale between the 2 populations: “I’m disappointed at times in my present accomplishments and think I should have accomplished much more.”^13^ Imposter phenomenon was associated with increased burnout,^13^ suicidal ideation,^13^ decreased professional fullfillment,^13^ and lower problem-solving confidence.^13,14^ Positive psychology–based coaching, per a 2020 literature review, involves identification, development, and utilization of strengths to turn a vision into reality.^15^ This style of coaching can help overcome imposter phenomenon which is a driver of physician burnout, mental illness, and negative self-perception.^15,16^ Professional development coaching, a form of peer coaching in which faculty from a different specialty coaches a resident using basic principles of positive psychology, has shown promise in fostering resident wellness without the use of significant resources.^17^

Appreciative inquiry, an asset-based approach to organizational changes, is another example of a positive psychology intervention for managing the stress associated with transition and change in health care systems. The 4-D model (discovery, dream, design, and destiny) of appreciative inquiry involves achieving goals by dreaming together as a team, focusing on strengths, and using those strengths to design strategies to achieve goals. This positive outlook at challenges can enhance meaning in work^18^ and augment professionalism and problem-solving capabilities of health care teams.^19^ Appreciative inquiry–centered narrative storytelling can also be used as a team-building exercise to cultivate reflective group learning, peer support, and meaningful professional relationships.^20^ Three Good Things is a simple journal activity in which participants log 3 good things that went well in a day. This well-being intervention was tested in health care workers for 15 days, and significant improvements from baseline were found in the emotional exhaustion, depression symptoms, and happiness metrics, with effect sizes of 0.55 to 1.57.^12^ In comparison, a Kirsch et al meta-analysis studying the effects of antidepressants found a difference between improvement in the drug and the placebo groups of only 0.32.^21^ Further, the results of the Three Good Things exercise were sustained at 6-month and 1-year intervals.^12^ Using this intervention qualitatively can also identify areas that provide meaning and joy to health care workers and give direction to leadership. This activity can also be conducted through cellular phone applications, making it more cost- and time-efficient than manual recording.

Mindfulness is the nonjudgmental observance of thoughts during overwhelming and stressful situations. Mindfulness-based interventions have shown promise for physicians in individual studies and meta-analyses.^22^ They are being used at several centers in the form of video modules, weekly activities, and group exercises, especially with resident physicians, and data suggest significant improvement in overall well-being.^22^

Given the alarming rise in physician burnout, with a 2015 study reporting that more than 50% of US physicians experience at least one symptom of burnout,^23^ action at the national, institutional, and personal levels is needed because physician burnout is directly related to substance abuse,^24^ suicidal ideation,^25^ self-reported medical errors,^26^ and overall poor quality of patient care.^26^ Although burnout is largely driven by work-related factors, including the clerical burden of electronic health records,^27^ long work hours,^28^ and work-home conflict,^29^ some of the interventions discussed here could be adopted by institutions and physician groups as a self-help measure to mitigate chronic workplace stress and improve overall mental health.
